# Temporal Dynamics of Motivation-Cognitive Control Interactions Revealed by High-Resolution Pupillometry

**DOI:** 10.3389/fpsyg.2013.00015

**Published:** 2013-01-29

**Authors:** Kimberly S. Chiew, Todd S. Braver

**Affiliations:** ^1^Department of Psychology, Washington University in St. LouisSt. Louis, MO, USA

**Keywords:** motivation, cognitive control, reward, pupillometry, incentive

## Abstract

Motivational manipulations, such as the presence of performance-contingent reward incentives, can have substantial influences on cognitive control. Previous evidence suggests that reward incentives may enhance cognitive performance specifically through increased preparatory, or proactive, control processes. The present study examined reward influences on cognitive control dynamics in the AX-Continuous Performance Task (AX-CPT), using high-resolution pupillometry. In the AX-CPT, contextual cues must be actively maintained over a delay in order to appropriately respond to ambiguous target probes. A key feature of the task is that it permits dissociable characterization of preparatory, proactive control processes (i.e., utilization of context) and reactive control processes (i.e., target-evoked interference resolution). Task performance profiles suggested that reward incentives enhanced proactive control (context utilization). Critically, pupil dilation was also increased on reward incentive trials during context maintenance periods, suggesting trial-specific shifts in proactive control, particularly when context cues indicated the need to overcome the dominant target response bias. Reward incentives had both transient (i.e., trial-by-trial) and sustained (i.e., block-based) effects on pupil dilation, which may reflect distinct underlying processes. The transient pupillary effects were present even when comparing against trials matched in task performance, suggesting a unique motivational influence of reward incentives. These results suggest that pupillometry may be a useful technique for investigating reward motivational signals and their dynamic influence on cognitive control.

## Introduction

*Cognitive control* is a general term used to describe mechanisms of active maintenance, attentional selection, and inhibition underlying higher cognition and making execution of adaptive, goal-oriented behavior possible. Motivational salience is a major factor that influences the goal selection and goal-related behavior – in daily life, we prioritize the goals that have the highest reward value. Accordingly, in recent years, there has been a surge of interest in research investigations focusing on motivational influences over cognitive control (Pessoa, 2009). Such investigations have been particularly important as increasing evidence suggests that increased motivation may improve task performance specifically through enhanced cognitive control (as opposed to a more general arousal effect; Pochon et al., 2002; Locke and Braver, 2008; Savine et al., 2010; Padmala and Pessoa, 2011). More specifically, some studies suggest that motivational incentives may change cognitive control processes by altering their temporal dynamics, increasing use of proactive (i.e., preparatory and/or sustained) control mechanisms, as opposed to reactive control mechanisms, which are engaged only as needed (Locke and Braver, 2008; Savine and Braver, 2010, 2012). Despite these suggestions, prior research on motivation-cognition interactions has not fully delineated the time courses of these interactions.

The present study aimed to rectify this by using high-resolution pupillometry to examine changes in the temporal dynamics of cognitive control (as indexed by pupil diameter) under motivational manipulations (i.e., the presence vs. absence of reward incentives). Pupil diameter is a well-established index of fairly specific changes in cognitive demand and effort (Beatty, 1982a,b; Granholm et al., 1996). Evidence for this was originally observed by Beatty and colleagues, who reported increasing pupil diameter with increased memory load (Kahneman and Beatty, 1966) and arithmetic demands (Ahern and Beatty, 1979). More recently, interest in pupillometry as a high temporal-resolution measure of cognitive control has been growing, following work that suggests that it may index changes in cognitive control dynamics related to typical development (Chatham et al., 2009) and decision-making (Satterthwaite et al., 2007). In addition, pupil diameter has also been shown to be responsive to emotional arousal associated with sympathetic nervous system activity (Bradley et al., 2008). This joint sensitivity to affective and cognitive influences suggests that pupillometric methods may be ideal for investigating the dynamics of motivation-cognition interactions; accordingly, confirming the validity of pupillometry for this purpose was a primary goal of the present study.

Following Locke and Braver (2008), we chose to investigate cognitive control dynamics in the present study using the AX-Continuous Performance Task (AX-CPT; Cohen and Servan-Schreiber, 1992; Servan-Schreiber et al., 1996; Braver et al., 2001). The AX-CPT is a context processing task that permits separate indices of proactive and reactive control. In the task, trials are composed of contextual cues immediately followed by response probes; target responses are required only when a specific cue-probe combination occurs (i.e., A followed by X; AX); otherwise, a different, non-target response is required. Because the target combination occurs with high-frequency (70%), it produces both a strong preparatory attentional expectancy triggered by the contextual cues (A = target; non-A [“B”] = non-target) and a target response bias associated with the X probe. Thus, utilization of proactive/preparatory control can be indexed on AY lure trials (Y = non-X probes); stronger interference on these trials (in terms of higher errors and slower performance) can be interpreted as reflecting the activation of a strong preparatory attentional expectancy. Conversely, reactive control can be indexed on BX lure trials in terms of the tendency to exhibit stimulus-triggered (i.e., probe-related) interference triggered by the presence of the X probe. Relative performance on AY vs. BX trials thus provides a marker of whether proactive or reactive control is dominant in AX-CPT task performance.

We predicted that we would replicate Locke and Braver’s (2008) results with respect to changes in task performance as a result of motivational incentives (overall enhanced performance, but additionally, observing greater AY interference and lessened BX interference, reflecting a shift to relatively greater proactive control). Additionally, we hypothesized that the presence of motivational incentives would result in greater transient pupil dilation (consistent with its putative role as an index of mental effort), and that this dilation increase would be triggered by the contextual cue (A or B) and then peak during the delay phase, prior to probe onset (i.e., in a preparatory fashion). Critically, we predicted that such transient, cue-evoked pupillary effects would be increased on incentive trials (relative to non-incentive trials), reflecting an increase in proactive control (i.e., increased preparatory attention toward the upcoming probe and associated response, based on contextual information).

In the current study, we utilized a mixed block/event experimental design to examine motivational incentive effects (Savine et al., 2010; Savine and Braver, 2012). In this design, participants perform separate baseline (no incentives offered) and reward blocks; within the reward block, non-incentive trials are randomly intermixed with incentive trials. The advantage of this design is that it allows examination of both trial-based (contrasting incentive and non-incentive trials within the reward block) and block-based (contrasting trials of the baseline block with non-incentive trials in the reward block) effects of incentive on task performance. Prior evidence suggests that these two types of effects may represent distinct transient and sustained (i.e., block-based) motivational influences on cognitive control (Jimura et al., 2010; Savine et al., 2010; Savine and Braver, 2012). Thus, we were also interested in examining whether motivational influences would be observed not only in transient (trial-evoked) increases in pupil dilation, but also in terms of sustained (tonic) effects.

Interestingly, prior research has suggested that transient and sustained components of pupil activity may index independent changes in control state, as predicted by the adaptive gain theory of locus coeruleus function (Gilzenrat et al., 2010). Adaptive gain theory posits that control depends on the balance between exploration and exploitation in pursuit of rewards, and that these states relate to tonic and phasic norepinephrine (NE) release, respectively (Aston-Jones and Cohen, 2005). In recent work, Gilzenrat et al. (2010) reported findings suggesting that sustained and transient pupil activity reflects these distinct components of NE release. Specifically, they argued that reduced tonic/increased phasic pupil activity related to exploitation and task engagement, while increased tonic/reduced phasic pupil activity related to exploration and task disengagement. The present task design allowed us to examine whether tonic and phasic pupil activity were inversely correlated (as they were in the Gilzenrat et al. results) and whether tonic and phasic activity dynamics shifted as a function of incentive. If reward motivation was associated with increased task engagement, and thus more exploitation, then greater phasic pupil activity and reduced tonic activity would be expected. However, if in this task paradigm, tonic pupil responses reflect other sustained, incentive-related processes besides exploration (e.g., arousal or increased effort) then different patterns might be present. The current study provided an opportunity to provide a first investigation of these two potentially distinct components of pupillary response.

## Materials and Methods

### Participants

Forty-seven healthy young adult participants took part (35 female; mean age 20.6 years ± 0.31). Participants were recruited from participant pools maintained by the Department of Psychology at Washington University in St. Louis. All participants were right-handed, had corrected-to-normal vision, and were free from psychiatric or neurological disorders. Informed consent was obtained from all subjects prior to participation, in accordance with the human subjects guidelines established by Washington University. Participants performed the experiment for a $10/hour payment, plus an additional monetary bonus due to reward incentives. Although participants were not informed of this until the end of the experiment, the bonus was a fixed amount ($5).

Fourteen participants were eliminated from analysis for missing 20% or more of their pupil data over the course of the experiment. This yielded 33 participants (25 female, mean age 20.3 years ± 0.30) who were included in the primary analyses reported below.

### Task paradigm

The AX-CPT consists of a series of continuous trials in which single letters are presented as cue-probe sequences. One specific cue-probe trial sequence requires a target response (i.e., “A” followed by “X”; AX trial), with all other combinations requiring a non-target response. The AX target trial-type occurs with 70% frequency, and is randomly intermixed with three types of non-target trials, each occurring with 10% frequency: AY (target cue, non-target probe), BX (non-target cue, target probe), and BY (non-target cue, non-target probe). Besides A and X, the stimuli that were used as “B” and “Y” stimuli were the letters B, D, E, F, G, M, P, S, U, Y, and Z.

**Figure 1:**
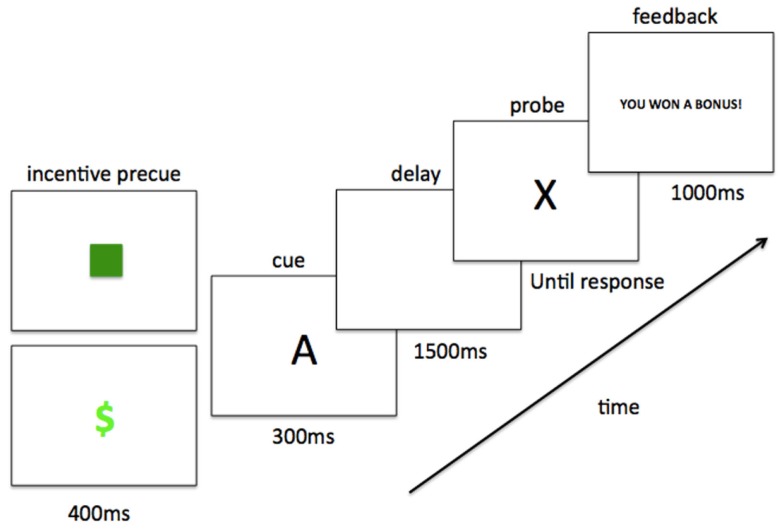
**Trial structure with timing**. Example AX (target) trial, with both non-incentive (green square) and incentive (green dollar sign) precues shown. One of these two precues preceded each contextual cue-probe letter pair and, in the reward block, indicated the presence/absence of incentive value for the trial.

Task trials consisted of the following structure (see Figure 1). The trial began with a 400 ms reward incentive precue; either a green square or a green dollar sign, presented centrally on a black screen. In the baseline block, participants were told to ignore these precues; in the reward block, participants were informed that they signified non-incentive and incentive trials, respectively. Following the incentive precue, the contextual cue (e.g., “A”) appeared for 300 ms, presented centrally in white on a black screen (Arial font, size 42). The contextual cue was followed by a 1500 ms fixation cross, and then a probe letter appeared in the same font (target probe was “X”). The probe remained on the screen until the participant responded to the cue-probe pair (target response for AX, and non-target response for all other combinations). Following a 250 ms delay, a feedback screen appeared for 1000 ms. In the baseline block and in non-incentive trials within the reward block, the feedback message read “Trial Over” if the participant had answered correctly and “Error” if the participant had answered incorrectly. In incentive trials within the reward block, the feedback message read “You Won a Bonus!” if the participant had replied accurately and under reaction time (RT) cutoff (i.e., meeting reward criteria), “Trial Over” if the participant had replied accurately but slower than RT cutoff, and “Error” if the participant had made an error. Each participant’s RT cutoff for reward receipt was individually determined from baseline block performance (explained further in Procedure below). Trials were separated by an inter-trial-interval (ITI) of 250 ms (15 participants) or 4000 ms (18 participants).

### Procedure

The experiment was visually presented using E-prime software (Psychology Software Tools, Pittsburgh, PA, USA) on a Dell PC computer. Participants were seated with a headrest to minimize head motion and viewed the paradigm on a computer monitor. Accuracy and RT data were collected via button press using an E-prime serial response box connected to the stimuli computer. Participants performed two blocks of the AX-CPT, always in a fixed order: a baseline block followed by a reward block. Each block consisted of 200 trials (140 AX target trials and 20 trials each of AY, BX, and BY non-targets, randomly intermixed). The baseline block of the task was performed without any knowledge of incentives, while in the reward block participants were told that they had the potential to win monetary incentives on some trials (up to $5 in addition to their participation compensation). Incentive trials were randomly intermixed with non-incentive trials (100 trials each) in the reward block, and were specified by the pre-trial incentive cue (square or dollar sign). On incentive trials within the reward block, participants were rewarded if they were accurate and faster than an individualized RT cutoff (calculated as the fastest 30th percentile of correct baseline block RTs). Reward information was provided by a visual feedback message, as described above.

### Pupillometry data collection

Pupil data were collected as participants completed the task using an Eyelink 1000 infrared eye tracker (SR Research Ltd., Mississauga, ON, Canada) running Eyelink software (version 4.48), sampling at 1000 Hz and at spatial resolution <0.01° RMS. Calibration and validation of gaze direction were conducted before each experimental run. Pupillometry data were preprocessed using in-house software written in Java (Oracle Corporation, Redwood Shores, CA, USA). Participants with more than 20% pupil data missing over the course of the experiment were discarded (14 participants). Blinks were corrected for using linear interpolation. Only correct response trials were included for pupillometric analysis (there were too few errors to analyze separately). For examinations of transient (trial-related) effects, we examined each trial’s pupil activity, normalized as a percent change from a baseline period (100 ms of ITI prior to each trial onset), while for examinations of sustained (block-related) effects, we examined pupil activity in Eyelink’s scaled pupil diameter values rather than absolute sizes – scaled values generally range between 3000 and 7000 [corresponding approximately to 3–7 mm; following Marshall (2007)].

### Data analysis

Behavioral performance data was analyzed with separate repeated-measures ANOVAs conducted on error rates and median correct RTs as dependent variables. To examine the block-related incentive effect on performance, we conducted a 2 × 2 × 2 ANOVA on non-incentive trials with block (baseline, reward), contextual cue (A, B), and probe (X, Y) as within-subject factors. By including only non-incentive trials in this analysis, it enables a purer test of the block-based effect unconfounded by the specific effect of incentive trials. To examine the trial-related incentive effect on performance, we conducted a 2 × 2 × 2 ANOVA on trials within the reward block, with trial-type (incentive, non-incentive), contextual cue (A, B), and probe (X, Y) as within-subject factors. Since only reward block trials are included in this analysis, it enables an independent test of trial-based reward effects unconfounded by any block-related effects.

Analyses of pupil activity were conducted by averaging specific time-windows during the trial. For analyses of the sustained incentive effect, pupil activity was examined at a 200 ms ITI period just prior to each trial’s onset in order to examine tonic, rather than task-evoked, pupil activity as a function of incentive block. For analyses of transient incentive effects, magnitudes were calculated for a 250 ms period of interest within the trial: the time window just prior to probe onset (referred to as pre-probe onset, timepoints 1950–2200 ms).

Average magnitudes of pupil dilation from these time periods of interest were examined using repeated-measures ANOVA in analyses analogous to those described previously for behavioral performance data. However, because the transient incentive analyses examined a period prior to probe onset, the ANOVA excluded the probe factor (i.e., incentive trial and contextual cue were the only two factors), because prior to probe onset trial-type could not be classified. Similarly, because the sustained incentive analyses involved the time window prior to trial onset, it only included block (reward, baseline) as a factor.

## Results

Although the subjects in our dataset completed the AX-CPT paradigm at two different ITIs (*N* = 15 at 250 ms, and *N* = 18 at 4000 ms), the only significant effect of ITI was a relatively minor interaction in behavioral task performance data^1^. Given that no other significant main effects or interactions with ITI were observed in either the task performance or pupillometric data, the following results are presented for the combined dataset (*N* = 33) collapsing across ITI. Results reported separately for each ITI are presented in the Appendix.

### Behavioral performance measures

#### Global incentive effects

The incentive manipulation was successful in improving performance, as participants achieved above-criteria (i.e., rewarded) performance on 78.5% of trials (range: 50–96%), vs. the expected rate of 30% reward if performance had remained at baseline levels.

#### Block-based incentive effects

**Figure 2:**
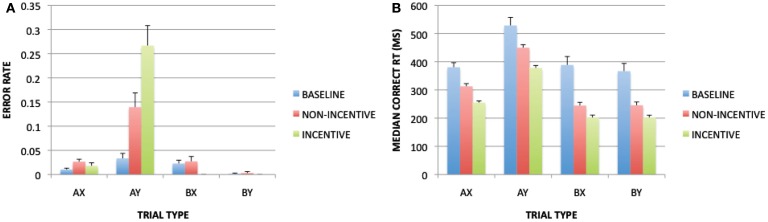
**Block-related (baseline vs. non-incentive trials) and trial-type (non-incentive vs. incentive trials) effects on task performance: (A) with error rates as a dependent measure; (B) with RTs as a dependent measure**.

The error rate ANOVA revealed a main effect of block [*F*(1, 32) = 27.339, *p* < 0.001], a main effect of cue [*F*(1, 32) = 12.851, *p* < 0.001], a main effect of probe [*F*(1, 32) = 4.383, *p* = 0.044], and significant interactions of block × cue [*F*(1, 32) = 20.177, *p* < 0.001], block × probe [*F*(1, 32) = 10.627, *p* = 0.0032], cue × probe [*F*(1, 32) = 22.753, *p* < 0.001], and block × cue × probe [*F*(1, 32) = 12.650, *p* = 0.001]. These effects were due to higher error rates in the reward block compared to the baseline block for AX trials and AY trials, but not for BX and BY trials (Figure 2A). This pattern closely replicates prior work where increased AX and AY trial errors were observed under incentive (Locke and Braver, 2008), and is consistent with a shift toward proactive control, since preparatory utilization of context cue information should benefit B-trial performance to a greater degree than A-trials, given that the A-cue does not unambiguously predict the upcoming probe or response. The ANOVA on RT yielded significant main effects of block [*F*(1, 32) = 29.435, *p* < 0.001], cue [*F*(1, 32) = 133.204, *p* < 0.001], and probe [*F*(1, 32) = 129.219, *p* < 0.001], as well as significant interactions of block × cue [*F*(1, 32) = 13.176, *p* = 0.001] and cue × probe [*F*(1, 32) = 144.468, *p* < 0.001]. The RT pattern was similar to that observed for error rate, in that RTs were faster in the reward block compared to baseline, with larger effects on B-cue than A-cue trials, and slowest performance on AY trials (again reflecting a proactive control bias; see Figure 2B).

#### Practice effects

Given the fixed order of baseline and reward blocks, a potential concern is whether the block-based effects may have actually reflected practice-related changes in performance rather than the influence of reward motivation. We believe this unlikely for a number of reasons. First, our prior studies have shown no evidence of practice contributing to block-based incentive effects (Savine et al., 2010), including recent work that used a more systematic design to directly control for such effects (involving post-incentive baselines and a no-incentive control group; Savine and Braver, 2012). Second, in a supplementary analysis of this dataset, in which each block was broken down into four 50-trial epochs, potential practice effects were found to disappear after the first epoch, while a clear discontinuity in performance was observed when comparing the last epoch of the baseline block to the first of the reward block. Details of this analysis are provided in the Appendix.

#### Trial-based incentive effects

For trial-type, the error rate ANOVA revealed significant main effects of incentive [*F*(1, 32) = 5.846, *p* = 0.021], cue [*F*(1, 32) = 36.803, *p* < 0.001], and probe [*F*(1, 32) = 29.219, *p* < 0.001], as well as significant interactions of incentive × cue [*F*(1, 32) = 16.761, *p* < 0.001], incentive × probe [*F*(1, 32) = 18.933, *p* < 0.001], cue × probe [*F*(1, 32) = 38.625, *p* < 0.001], and incentive × cue × probe [*F*(1, 32) = 11.439, *p* = 0.002]. These effects reflected a pattern in which, on incentive trials, error rates were lower (and nearly eliminated) for all trials except for AY, which was strongly increased. The RT ANOVA yielded main effects of incentive [*F*(1, 32) = 73.196, *p* < 0.001], cue [*F*(1, 32) = 316.687, *p* < 0.001], and probe [*F*(1, 32) = 395.971, *p* < 0.001], as well as significant interactions of incentive × cue [*F*(1, 32) = 8.603, *p* = 0.006] and cue × probe [*F*(1, 32) = 283.264, *p* < 0.001]. These effects reflected that incentive trials were associated with faster RTs for all trial-types, but that the speeding was greater for A-cue than B-cue trials. This may have reflected the fact that RTs on B-cue trials were at floor levels. Indeed, the contrast of near-optimal performance on BX trials (0% errors, ∼200 ms RT) and much poorer performance on AY trials (∼25% errors, RTs double that of BX) suggest that performance had almost completely shifted toward proactive control and away from reactive control on incentive trials.

#### Speed-accuracy tradeoff effects

Another potential concern is whether incentive effects reflect primarily a speed-accuracy tradeoff (SAT) rather than a motivation-based enhancement of proactive control. This was an important concern, as a general trend of higher error rates and decreased RTs was observed in the reward block relative to baseline. We addressed this issue by examining SAT directly for both block-based and trial-based incentive effects, by correlating the change in RT against the change in error rate (baseline vs. non-incentive trials, and non-incentive vs. incentive contrast) across participants. None of the correlations reached significance, suggesting that the block-based and incentive-based patterns were not strongly reflective of a SAT effect. Next we looked at SATs within AY trials, where apparent SAT effects seemed most prominent, by correlating error rates with RTs within each condition (baseline, non-incentive and incentive) separately. Indeed, AY errors and RTs were correlated within each of the conditions, but most strongly correlated on incentive trials (in baseline trials: *r* = −0.321, *p* = 0.07; in non-incentive trials, *r* = −0.417, *p* = 0.016; in incentive trials, *r* = −0.633, *p* < 0.001). This pattern of increased SAT with incentive in AY trials is actually highly consistent with our theoretical interpretation of increased proactive control. Specifically, AY trials do not benefit from increased proactive control (i.e., enhanced cue-related preparation toward the upcoming probe). This is because strong preparation of an expected target response following an A-cue should lead to an increased tendency for interference when a Y probe appears. The pattern suggests that the participants most likely to make a quick, preparation-based target response following probe onset were also the ones most likely to have the highest AY error rate.

### Pupillometry measures

#### Sustained incentive effects

**Figure 3:**
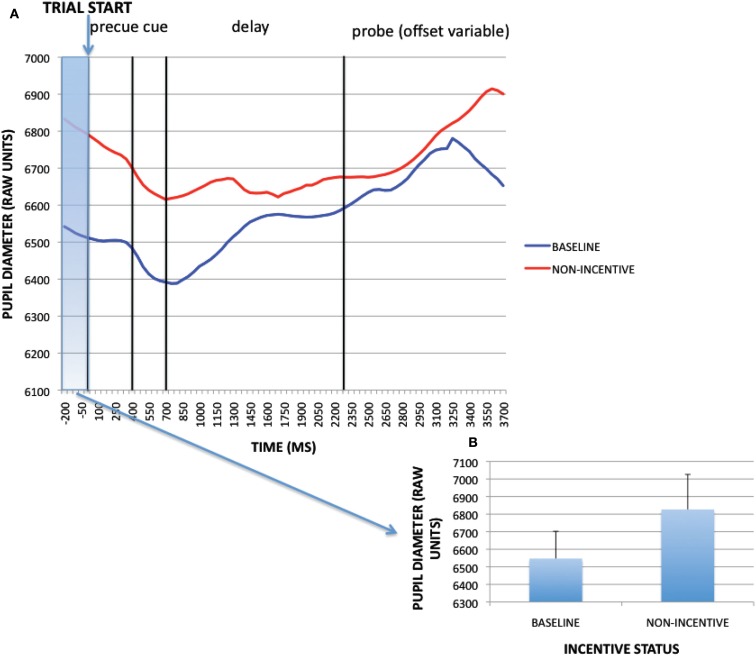
**(A)** Pupil timecourses as a function of incentive status for the sustained incentive contrast (baseline vs. non-incentive trials, averaged across trial-types). **(B)** Sustained incentive effects (as averaged pupil magnitudes) at pre-trial period (−200 to 0 ms).

A paired-samples *t-*test was conducted on the pre-trial time window (−200 to 0 ms) to examine the effect of block (reward, baseline) on pupil dilation. The effect of block was significant [*t*(32) = −3.049, *p* = 0.005]: pupil dilation was greater in the reward block than in baseline. Baseline timecourses and non-incentive timecourses within the reward block (averaged across trial-type) and averaged magnitudes for the pre-trial period of interest for the sustained incentive contrast are shown in Figure 3. Visual examination of these timecourses verifies that not only was pupil dilation greater in the reward block during the pre-trial period, but also that this difference was present throughout the course of the trial as well. This pattern is suggestive of a tonic increase in cognitive effort on reward block trials compared to baseline block trials. Similar to the supplementary analyses conducted above, we also tested whether such block-based pupillary effects could be the result of practice or time-on-task, by breaking down the data for each block into four 50-trial epochs. As reported more fully in Appendix, practice effects dissipated within the first epoch of the baseline block, while a clear discontinuity in pupil dilation was observed between the last epoch of baseline and the first epoch of the reward block. Thus, the tonic pupil effects appear to be a result of motivational influences rather than practice or time-on-task.

#### Transient incentive effects

**Figure 4:**
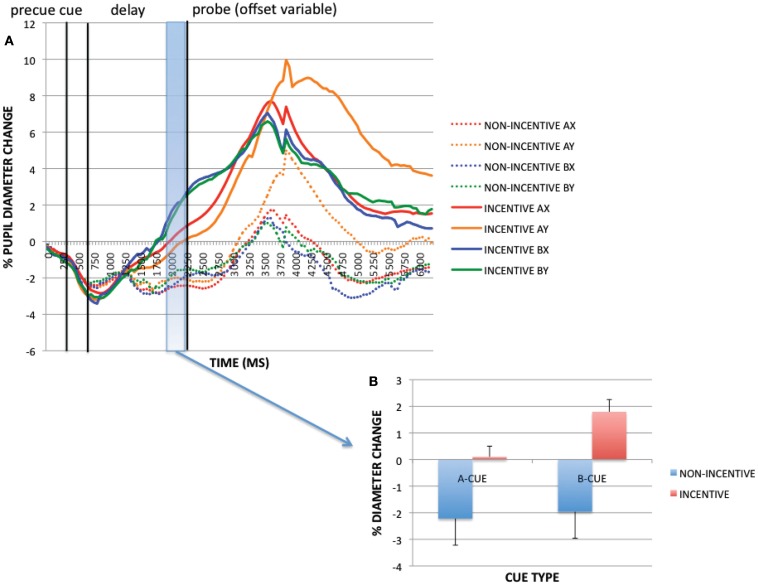
**(A)** Pupil trial timecourses as a function of incentive status and trial for the incentive cue contrast. **(B)** Incentive trial effects (as averaged pupil magnitudes) at pre-probe onset period (1950–2200 ms).

To examine trial-evoked incentive effects, we compared incentive and non-incentive trials at a 250 ms time window, during pre-probe onset (1950–2200 ms). Within this period, the ANOVA revealed a significant main effect of incentive on pupil dilation [*F*(1, 32) = 50.192, *p* < 0.001; see Figure 4], with greater pupil dilation on incentive vs. non-incentive trials. Additionally, a main effect of cue [*F*(1, 32) = 13.438, *p* = 0.001], and an incentive × cue interaction [*F*(1, 32) = 7.678, *p* = 0.009] were found. These latter effects reflected greater dilation on B-cue compared to A-cue trials, but selectively on incentive trials. With pupil dilation as a putative marker of mental effort, this pattern of results suggests that greater effort may be exerted with incentive – and notably – in B-cue trials relative to A-cue trials. This B > A pattern is intriguing, because it suggests that greater preparatory effort is exerted in non-target trials relative to target trials, possibly due to the utility of the contextual cue, which on B-cue trials indicates the need to overcome the dominant target response bias (i.e., target responses are made on 70% of all trials, and on 87.5% of A-cue trials, but 0% of B-cue trials).

#### Sustained vs. transient effects

**Figure 5:**
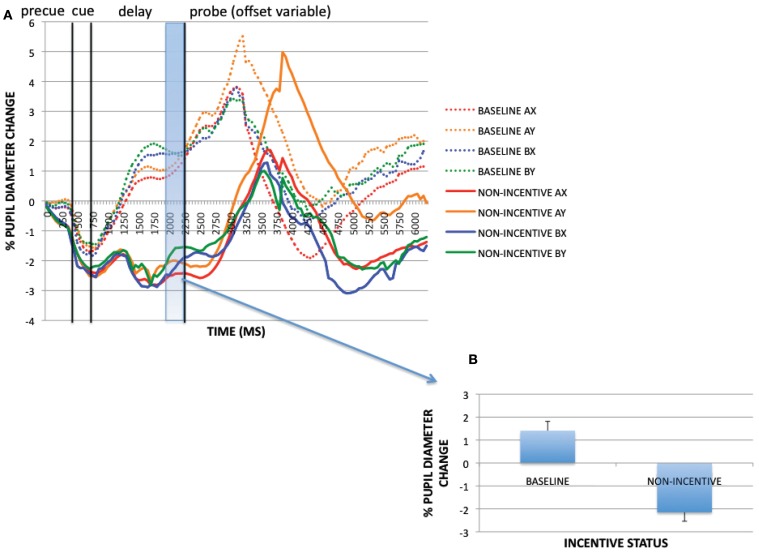
**(A)** Trial-evoked (normalized) pupil timecourses in baseline and non-incentive trials within the reward block. **(B)** Trial-evoked pupil activity (as averaged pupil magnitudes) in baseline and non-incentive conditions at pre-probe onset period (1950–2200 ms).

The preceding analyses utilized non-normalized data taken from the pre-trial period to identify sustained (block-based) incentive effects, and normalized data at the pre-probe period (along with the cue-period) to identify transient (trial-evoked) incentive effects. Yet it is possible that the sustained effects of incentive also interacted with the transient effects. To examine this possibility, we compared the trial-evoked pupil activity in baseline block trials and non-incentive trials within the reward block, after normalization, to identify any transient activation patterns that might occur in the context of a sustained incentive effect. Visual inspection of the normalized pupil time courses indicates that *less* trial-evoked dilation was observed in non-incentive compared to baseline trials (see Figure 5). This was confirmed by analyzing averaged pupil magnitudes (all trials collapsed) at the pre-probe onset period [1950–2200 ms; *t*(32) = 8.646, *p* < 0.001]. Thus, while the reward block was associated with increased tonic pupil dilation, it also appears that reduced transient dilation was also present relative to baseline, specifically on non-incentive trials. In other words, performance in the baseline block could be characterized by relatively low tonic pupil activity but relatively high phasic activity, while performance in non-incentive trials within the reward block might be characterized by relatively high tonic activity but lower phasic activity (potentially as a result of the reduced trial-based effort demands caused by the sustained incentive-related enhancement in proactive control). In contrast, performance on incentive trials within the reward block may be characterized by both relatively high tonic and phasic pupil activity.

#### RT effects

A potential confound associated with the analyses of incentive effects on pupil dilation is that RTs on incentive trials were globally faster than non-incentive (as well as baseline block trials). If RT serves as an index of the degree of mental effort exerted on a given trial, then it is hard to disentangle whether the increase in pupil dilation reflects an increase in cognitive effort *per se*, the motivational effect of incentive, or both. To attempt to disentangle these contributions, we examined pupil dilation as a function of RT, both on incentive and non-incentive trials within the reward block, subdividing each according to whether RT was faster or slower than the individual’s RT cutoff for receiving reward. Importantly, the application of the same cutoff to non-incentive trials (even though the cutoff was meaningless to the participant on these trials), as well as incentive trials, appropriately isolates the subset of non-incentive trials that putatively reflect a high degree of mental effort (because they are associated with a fast RT). Finally, the analysis was conducted only on AX trials, since these were the most frequent, in order to eliminate trial-type related differences in pupil dilation and RT.

**Figure 6:**
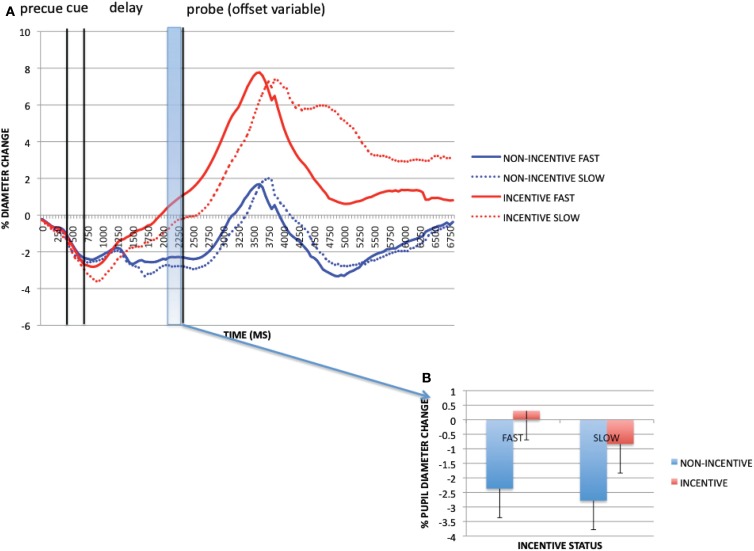
**(A)** Reward block pupil timecourses (correct AX trials only) split by incentive and reaction time (faster/slower than RT criterion) for each participant. **(B)** Incentive trial and RT effects at pre-probe onset period (1950–2200 ms). These data reveal that pupil dilation is associated with both task performance and incentive status, and that these effects do not appear to interact.

Figure 6 shows the pupil dilation time-course data as a function of both incentive (incentive vs. non-incentive trials) and RT (faster vs. slower than cutoff). Visual inspection reveals potential effects of both of these factors: greater pupil dilation is observed on fast RT trials compared to slow RT, and on incentive trials compared to non-incentive. This pattern was formally tested in the pre-probe time window of primary interest (1950–2200 ms) in a 2 × 2 ANOVA (Figure 6B). A trend effect of RT was observed [*F*(1, 32) = 3.639, *p* = 0.065; fast trials dilated more than slow], as well as a significant incentive effect [*F*(1, 32) = 33.308, *p* < 0.001]. However, the two factors did not interact [*F*(1, 32) = 0.878, *p* = 0.356]. Moreover, the effect of incentive was significantly stronger than the effect of RT. The key comparison of interest is that of slow incentive trials vs. fast non-incentive trials, since by definition, in this contrast, non-incentive trials are associated with better objective performance (faster RTs, equivalent accuracy) than incentive trials. Yet, even for this comparison, dilation was still significantly greater on incentive trials [*t*(32) = 3.069, *p* = 0.004]. Thus, although pupil dilation has been considered an index of mental effort, this examination makes clear its sensitivity to motivational influences occurs beyond simple association with overt task performance.

## Discussion

The present study used high-resolution pupillometry to examine effects of reward motivational incentives during performance of a cognitive control task. Previous evidence suggests that incentives generally enhance cognitive control performance, specifically by shifting performance into a more proactive mode, characterized by enhanced preparatory processing. We were able to test this hypothesis using pupil dilation as an indirect measure of preparatory cognitive effort in a rewarded version of the AX-CPT, a cognitive control task that permits relative examination of both proactive and reactive control.

Results from the present study were highly consistent with this hypothesis. Incentive was associated with enhanced task performance overall, both in terms of block-based and trial-related effects, and this result appeared to be independent of practice effects. However, performance on AY trials was worsened, consistent with increased proactive utilization of contextual cue information (which is misleading on AY trials). Indeed, this effect was maximally strong on incentive trials, for which AY performance was very poor (∼25% errors, slow RT) while performance of BX trials was near optimal (∼0% errors, 200 ms RT). Since BX performance is poor under conditions in which reactive, rather than proactive control is used (since the X probe is associated with a dominant target response bias), the combined performance pattern across the two trial-types suggests an almost complete shift toward proactive control and away from reactive control. Observation of an increasing SAT in AY trials with incentive, in terms of both block-based and trial-based effects, was also consistent with the performance profile of a shift toward proactive control, i.e., the participants that apparently most strongly relied upon proactive control showed the fastest RTs but also the highest error rates.

Reward motivational incentives were also associated with increased pupil dilation, both in terms of sustained and transient components. Importantly, when examining transient effects, the effect was clearly present prior to probe onset (and thus, response execution). This pattern provides strongly convergent data that incentive-related mechanisms of performance enhancement were operating in a preparatory or proactive fashion. As such, the results clearly validate pupillometric indices as predictive markers of incentive-related changes in cognitive control dynamics, prior to and independent from overt responding.

Another important finding of this study was that trial-by-trial increases in pupil dilation were observed to occur to a greater extent on B-cue trials than A-cue trials. This pattern of activity is similar to patterns of activity observed in neuroimaging studies of the AX-CPT, where increased activity in lateral prefrontal cortex (PFC) has also been observed on B-cue trials (Paxton et al., 2008; Edwards et al., 2010). This pattern has been typically interpreted as reflecting the increased need for preparatory cognitive control following B-cues, since these indicate that the dominant target response bias to the upcoming probe will need to be suppressed (MacDonald III and Carter, 2003). Specifically, target probe responses occur with high-frequency in general (70 vs. 30%) and following A-cues (87.5 vs. 12.5%), but should never be made following B-cues. Thus, the increase in pupil dilation and lateral PFC activity could reflect the engagement of these preparatory control processes. However, other interpretations cannot be ruled out. In the classic AX-CPT, B-cues also occur much less frequently than A-cues (20 vs. 80%), so the greater pupil dilation could be a more general effect of the novelty or surprise associated with this cue-type. Finally, a third interpretation is that B-cues are also more informative than A-cues for response preparation, in that they have greater predictive validity (target responses are 87.5% valid after A-cues, while non-target responses are 100% valid after B-cues). Variants of the AX-CPT paradigm have been developed that control for differential frequency and/or predictive validity of A and B-cues (i.e., Locke and Braver, 2008; Richmond et al., 2012). These could be used in future investigations to more fully clarify the source of this pattern of pupil activity.

How these influences may manifest in terms of neuromodulatory activity affecting dilation remains to be clarified. The sensitivity of the pupil as an index of the locus coeruleus-norepinephrine (LC-NE) system is well-documented (Aston-Jones and Cohen, 2005; Gilzenrat et al., 2010; Murphy et al., 2011). Our data are somewhat consistent with evidence from Gilzenrat and colleagues that tonic and phasic pupil activity dynamics may be inversely correlated (given our observation of high phasic/low tonic activity in baseline trials, vs. low phasic/high tonic activity in non-incentive trials within the reward block). Interestingly, adaptive gain theory predicts that a pattern of low phasic/high tonic pupil activity should be predictive of increased task exploration and decreased task engagement. Although the high tonic/low phasic pattern was observed in non-incentive trials within the reward block, it was associated with a behavioral shift toward enhanced proactive control (i.e., increased task engagement) relative to baseline performance. Further, incentive trials within the reward block were characterized by high tonic *and* phasic pupil activity, and were associated with further behavioral shifting toward enhanced proactive control relative to both non-incentive and baseline trials. Given that adaptive gain theory predicts that high phasic/low tonic pupil and LC-NE activity should characterize motivated performance (i.e., enhanced task engagement), how this pattern of pupil activity might correspond to control state is not yet clear, and may reflect other possible influences on pupil dilation in addition to LC-NE system activity. For example, the processing of reward incentives such as those used in the present study has been associated with dopaminergic (DA) activity. While, to our knowledge, pupil dilation has not been directly associated with DA activity, converging evidence from the present study (suggesting that pupil diameter is sensitive to incentive value) and from previous neuroimaging work (suggesting that DA midbrain areas index incentive value in a rewarded cognitive task; Savine and Braver (2010) raise interesting possibilities that pupil dilation may be sensitive to more complex interactions of multiple neurotransmitter systems.

Given these possibilities, using pupillometry to examine changes in control dynamics as a result of motivational/affective manipulations may also assist in characterizing such motivational and affective factors more generally. One of the key findings from this study appears to be highly relevant for this issue. Specifically, we observed that pupil dilation was greater in slow incentive trials compared to fast non-incentive trials within the reward block. This suggests that dilation is not merely a direct reflection of effort related to performance; it may also be subject to influences such as motivational salience and emotional arousal that are not directly associated with overt task performance. Other prior work is also suggestive that pupil dilation is sensitive to emotional arousal, independent of cognitive demand (Bradley et al., 2008). Yet it is still unclear whether changes in pupil dilation manifest in the same way for motivational salience as for emotional arousal.

How emotion and motivation are characterized in terms of their relative influences on cognitive control is a question of growing interest (e.g., Chiew and Braver, 2011), especially in light of previous studies suggesting that positive affect and reward may have differing effects. For example, in a prior study of AX-CPT performance under positive affect conditions, an enhancement of reactive control was observed (Dreisbach, 2006). This pattern contrasts strongly with the findings of both this study, and our prior work using fMRI (Locke and Braver, 2008), in which reward motivation led to an enhancement of proactive control during AX-CPT performance. Positive affect has also been associated with reduced cue use in other cueing paradigms, including response priming and cued task-switching (consistent with a shift away from proactive control; Frober and Dreisbach, 2012), and may reduce conflict adaptation (van Steenbergen et al., 2009), in contrast to performance-contingent reward, which may increase sequential conflict adaptation (Braem et al., 2012). Pupillometry, as a measure that appears to be sensitive to both affective and cognitive influences, may be an ideal tool to compare the dynamics of affect-cognition interactions and clarify how emotional and motivational influences on cognition may relate. Thus, an important future direction, given previous experimental observations of divergent affect and reward effects, would be to directly compare the effects of emotional arousal (e.g., through positively valenced cues) and motivational salience (e.g., through motivationally significant cues) during performance of a cognitive task such as the AX-CPT used here, to determine their relative effects on pupillometric indices.

Numerous other important research questions remain. The present study is intended as an initial investigation into the use of pupillometry in indexing cognitive control dynamics as a function of motivational manipulations. Our results validate this approach, and suggest exciting future directions for the use of pupillometry in further characterizing motivation, affect, and their interactions with cognition. Given pupillometry’s utility in indexing cognitive and neuromodulatory dynamics at a high degree of temporal-resolution, and its relatively easy and inexpensive collection compared to other imaging or electrophysiological measures (i.e., fMRI, EEG, MEG), pupillometry may be an ideal candidate technique for preliminary investigations of cognitive phenomena (prior to using more expensive or invasive measures) or data collection concurrent with other methodologies. Another important direction to be addressed by future research is examining the influence of cognitive and reward-related individual differences in accounting for variability in task performance and pupil dilation. Behavioral and neuroimaging studies have already observed intriguing correlations between individual differences in incentive-related performance enhancement and reward-related personality traits (Savine et al., 2010) and cognitive control-related prefrontal brain activity (Jimura et al., 2010; Savine and Braver, 2010). Additionally, it has been suggested that individual differences in working memory span may influence the extent to which mental effort is exerted in an incentivized reading span task, and that this effort can be characterized using pupillometry (Heitz et al., 2008). It would be useful for future work in this domain to include large-sample designs that enable statistically well-powered measurements of individual differences in both affective and cognitive traits. Relating such differences to changes in task performance and pupil activity with cognitive, emotional, and motivational manipulations will permit us to gain a more nuanced picture of how these systems interact and the dynamics by which they do so.

## Conflict of Interest Statement

The authors declare that the research was conducted in the absence of any commercial or financial relationships that could be construed as a potential conflict of interest.

## Figures and Tables

**Figure A1:**
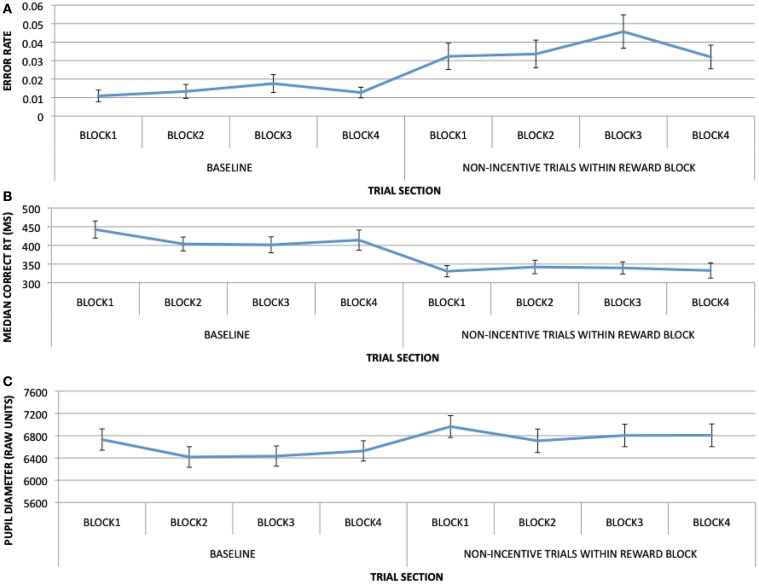
**Task performance and pupil data as a function of 50-trial sections, for baseline and non-incentive trials in the reward block: (A) with error rates as a dependent measure; (B) with RTs as a dependent measure; (C) with pupil dilation at pre-trial period (−200 to 0 ms) as a dependent measure**.

## Footnotes

^1^When examining the block-related incentive effect on task performance RTs (baseline vs. non-incentive trials), a significant ITI × probe interaction was observed (*p* = 0.032). This interaction was due to faster X-probe RTs at long vs. short ITI, vs. no significant difference in Y-probe RTs across ITI condition.

## Appendix

### Practice effects: Analyses

To clarify whether the block-based effects observed in the present study could be attributed to practice-related effects as opposed to reward motivation, we divided the baseline and reward blocks into four 50-trial periods each and examined task performance and pre-trial pupil magnitude (in the 200 ms prior to trial start) for baseline and non-incentive trials over the course of these periods (shown in Figure A1).

For performance, trial section was significant for baseline block RTs [*F*(3, 96) = 3.342, *p* = 0.022]: performance in the first 50 trials was significantly slower than in the subsequent second 50 (*p* = 0.007) or third 50 trials (*p* = 0.028), indicating a practice effect within the baseline block itself. No significant effects of trial section were otherwise observed. In contrast, RTs in the last 50 trials of the baseline block, compared to non-incentive trials in the first 50 trials of the reward block, showed a strongly significant effect [*t*(32) = 3.936, *p* < 0.001] owing to an abrupt decrease in RTs with the onset of the reward block. This effect was larger in magnitude than the practice effects in the baseline block, and occurred later in the experimental session, suggesting that it was likely as a result of incentive rather than practice. With performance as measured by error rates, trial section was not significant for baseline block [*F* < 1] or non-incentive trials within reward block [*F* < 1].

For pupil magnitude, trial section was significant for both baseline block [*F*(3, 96) = 6.398, *p* = 0.001) and non-incentive trials within reward block [*F*(3, 96) = 6.651, *p* < 0.001]. For baseline, this effect was driven by higher dilation in the first 50-trial section than in the second (*p* < 0.001), third (*p* = 0.005), or fourth (*p* = 0.026). For non-incentive trials within reward block, this effect was again driven by higher dilation in the first 50 trials than in the second (*p* < 0.001), third (*p* = 0.021), and fourth (*p* = 0.037), but also lower dilation in the second 50 trials vs. the third 50 trials (*p* = 0.054). When comparing pupil magnitudes in the last 50 trials of the baseline block to non-incentive trials of the first 50 trials of the reward block, dilation was significantly greater in the reward block than in baseline (*p* < 0.001). The overall pattern of pupil dilation from baseline to non-incentive trials within the reward block suggests practice effects within each block, but also an abrupt increase in dilation at the beginning of the reward block, relative to the end of the baseline block, that is more suggestive of incentive effects than gradual, practice-related change.

**Table A1:** **Behavioral performance measures: results split by ITI**.

Analysis	Effect examined	Results in short ITI data (*N* = 15)	Results in long ITI data (*N* = 18)	Significant difference between ITIs
Global incentive effects	Percentage of above-criteria (i.e., rewarded) incentive trials	78.9%	78.2%	N
Block-based incentive effects: errors	Error rates in baseline vs. non-incentive trials within reward block (Block × cue × probe ANOVA)	Significant effects: Block Block × cue Cue × probe	Significant effects: Block Cue Probe Block × cue Block × probe Cue × probe Block × cue × probe	N
Block-based incentive effects: RTs	RTs in baseline vs. non-incentive trials within reward block (Block × cue × probe ANOVA)	Significant effects: Block Cue Probe Block × cue Cue × probe	Significant effects: Block Cue Probe Block × cue Cue × probe	Y (Probe × ITI *p* = 0.026; See text Footnote 1)
Trial-based incentive effects: errors	Error rates in incentive vs. non-incentive trials within reward block (Incentive × cue × probe ANOVA)	Significant effects: Cue Probe Incentive × cue Incentive × probe Cue × probe Incentive × cue × probe	Significant effects: Incentive Cue Probe Incentive × cue Incentive × probe Cue × probe Incentive × cue × probe	N
Trial-based incentive effects: RTs	RTs in incentive vs. non-incentive trials within reward block (Incentive × cue × probe ANOVA)	Significant effects: Incentive Cue Probe Cue × probe	Significant effects: Incentive Cue Probe Incentive × cue Cue × probe	N
Speed-accuracy tradeoff effects	Correlations between error rates and RTs for AX, AY, and BX trials in baseline, non-incentive, and incentive conditions	No significant correlations found in short ITI data alone	Significant correlations: Incentive AX (*r* = −0.540, *p* = 0.021) Incentive AY (*r* = −0.726, *p* = 0.001)	N (correlations compared using Fisher *r* to *z* transformation)

**Table A2:** **Pupillometry measures: results split by ITI**.

Analysis	Effect examined	Results in short ITI data (*N* = 15)	Results in long ITI data (*N* = 18)	Significant difference between ITIs
Sustained incentive effects	Pupil magnitude during 200 ms pre-trial period in baseline vs. non-incentive trials within reward block (*t*-test)	Not significant	Greater pupil dilation in reward block trials vs. baseline (*p* = 0.003)	N
Transient incentive effects	Pupil magnitude during 250 ms pre-probe period in incentive vs. non-incentive trials within reward block (Incentive × cue ANOVA)	Significant effects: Incentive Cue Incentive × cue	Significant effects: Incentive Cue	N
Sustained vs. transient effects	Pupil magnitude during 250 ms pre-probe period in baseline vs. non-incentive trials within reward block (*t*-test)	Significant (Baseline > reward block transient activity)	Significant (Baseline > reward block transient activity)	N
RT effects	Pupil magnitude during 250 ms pre-probe period in fast/slow incentive vs. non-incentive trials (Incentive × speed ANOVA)	Significant effects: Incentive	Significant effects: Incentive Speed	N
